# First Report of Hepatitis E Virus Infection in Sika Deer in China

**DOI:** 10.1155/2015/502846

**Published:** 2015-04-09

**Authors:** Xiao-Xuan Zhang, Si-Yuan Qin, Yuan Zhang, Qing-Feng Meng, Jing Jiang, Gui-Lian Yang, Quan Zhao, Xing-Quan Zhu

**Affiliations:** ^1^College of Animal Science and Technology, Jilin Agricultural University, Changchun, Jilin 130118, China; ^2^State Key Laboratory of Veterinary Etiological Biology, Lanzhou Veterinary Research Institute, Chinese Academy of Agricultural Sciences, Lanzhou, Gansu 730046, China; ^3^Jilin Entry-Exit Inspection and Quarantine Bureau, Changchun, Jilin 130000, China; ^4^Jiangsu Co-Innovation Center for Prevention and Control of Important Animal Infectious Disease, Yangzhou, Jiangsu 225009, China

## Abstract

Hepatitis E virus (HEV), a single stranded RNA, nonenveloped virus, belongs to the genus *Hepevirus*, in the family of *Hepeviridae*. In this study, 46 (5.43%) out of the 847 serum samples from sika deer (*Cervus nippon*) were detected as seropositive with hepatitis E virus (HEV) by enzyme linked immunosorbent assay (ELISA). These samples were collected from Inner Mongolia and Jilin and Heilongjiang provinces in China, between October 2012 and October 2013. Seroprevalence of HEV infection in male and female deer was 4.82% and 6.52%, respectively. HEV seroprevalence in sika deer from different geographical locations varied from 3.13% to 6.73%. There was no significant difference in HEV seroprevalence between sika deer collected in autumn (5.65%) and winter (4.85%). This is the first report of HEV seroprevalence in sika deer in China, which will provide foundation information for estimating the effectiveness of future measures to control HEV infection in sika deer in China and assessing the potential risk of humans infected with HEV after consumption of undercooked or raw meat from infected sika deer.

## 1. Introduction

Viral hepatitis is a global health problem [1], especially hepatitis E. HEV is the causative agent of hepatitis E [2]. Hepatitis E virus (HEV), a single stranded RNA, nonenveloped virus, belongs to the genus* Hepevirus*, in the family of* Hepeviridae* [2–6]. Previous studies introduced four routes to transmission of HEV. They are fecal-oral transmission, foodborne transmission, transfusion, and vertical transmission [7]. HEV infection can lead to protracted coagulopathy and cholestatis and is also associated with death especially among pregnant women [7–10].

Hepatitis E was first recognized in India in 1978 [5]. Avian HEV and mammalian HEV are major species of hepatitis E virus [5]. More than four genotypes (genotypes 1, 2, 3, and 4) have been identified in HEV [6, 11, 12]; for example, genotypes 1 and 2 of HEV were commonly prevalent in developing countries, and genotype 2 appears to be exclusively anthroponotic, while genotype 1 infects mainly humans but has also been detected in pigs [13, 14]. Genotype 3 was distributed around the world and could cause severe HEV infection in humans and animals. Most of HEV genotype 4 infections were in Asian countries, and they have been found in humans and pigs [11, 15–18]. Recently, genotype 5 has been proposed as the designation for a variant of HEV isolated from wild boars [12]. Haqshenas et al. [19] and Huang et al. [20] identified a novel HEV strain, which was isolated from chicken, but Avian HEV could not infect nonhuman primates [20]. Humans can be infected by six genotypes of mammalian HEV, namely, types 1 to 4 and two additional genotypes from wild boar [5].

In view of such serious situation, a large number of investigations about HEV have been carried out worldwide [11–27]. In China, infection of HEV has also been reported in wild rats, rabbits, pigs, sheep, parrots, chickens, dogs, cats, and humans [3, 29–35], but no information was available about HEV infection in sika deer. The objectives of this survey were to investigate the distribution and seroprevalence of HEV infection in sika deer from 4 cities, Northern China, for the first time, provide the “baseline” data for the control of HEV infection, and assess the potential risk that people are infected by HEV.

## 2. Materials and Methods

### 2.1. Ethics Statement

This study was approved by the Animal Ethics Committee of Lanzhou Veterinary Research Institute, Chinese Academy of Agricultural Sciences (Approval number LVRIAEC2012-008). The sika deer, from which blood was collected, were handled in accordance with good animal practices required by the Animal Ethics Procedures and Guidelines of the People's Republic of China.

### 2.2. The Study Site

**Figure 1:**
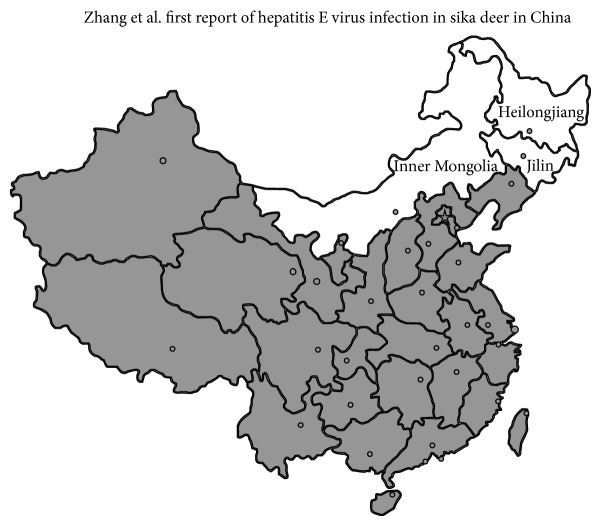
Map showing Heilongjiang province, Jilin province, and Inner Mongolia Autonomous Region stranded out in white color in northeastern China, where sika deer serum samples were collected for detection of hepatitis E virus antibodies.

The samples in this study were collected from 4 cities, Northern China (Figure 1). Changchun (43°05′~45°15′N, 124°18′~127°05′E) is the capital of Jilin province, and it is one of the central cities in Northeast China and belongs to a semiwet monsoon type zone. Jilin City (42°31′~44°40′N, 125°40′~127°56′E) is located in Jilin province, and the climate of Jilin City is northerly continental monsoon type. Harbin (44°04′~46°40′N, 125°42′~130°10′E) is one of the central cities in Northeast China and the capital of Heilongjiang province. Harbin belongs to a temperate continental monsoon type climate. Chifeng (41°17′10′′~45°24′15′′N, 116°21′07′′~120°58′52′′E) is located in the upper reaches of the Xiliao River. Chifeng has a local steppe climate. Sika deer are mostly originated in Changchun, Chifeng, and Harbin in Northern China.

### 2.3. Serum Samples

All the sika deer blood samples (208 from Harbin city, 316 from Changchun city, 163 from Jilin City, and 160 from Chifeng city) were randomly collected from the 4 cities, Northern China, between October 2012 and October 2013. All the sika deer were bred in the semifree range system. Samples were transported to the laboratory in the College of Animal Science and Technology, Jilin Agriculture University, Jilin province, China. They were kept at 37°C for 2 h and then at 4°C for 1 h, and then centrifugation was carried out at 1,000 ×g for 10 min, and the serum was separated and kept at −20°C until further analysis. Information of sika deer about seasons and geographic origin of sampling, gender, and age was recorded.

### 2.4. Serological Examination

Circulating antibodies (CAb) against HEV were tested by the species-independent double-antigen sandwich (das) ELISA using a commercially available kit (MP Biomedicals Asia Pacific Pte. Ltd., Singapore) according to the manufacturer's instructions [22]. Positive, negative, and blank controls were supplied in the kit and set in each test.

### 2.5. Statistical Analysis

The differences of seroprevalence of HEV infection in sika deer from different locations, gender, and seasons were analyzed statistically using SAS software (version 9.1, SAS Institute, Inc., Cary, NC) [36]. Results were considered statistically significant if *P* < 0.05. Odds ratios (OR) and their 95% confidence interval (95% CI) were provided in this study.

## 3. Results

A total of 46 (5.43%, 95% confidence interval (CI) 3.91–6.96) out of 847 sika deer were detected as seropositive of HEV by ELISA (Table 1). Seroprevalence of HEV in male deer was 4.82% (95% CI 3.01–6.62), which was lower than that in female deer (6.52%, 95% CI 3.75–9.28), but the difference was not statistically significant (*P* > 0.05). HEV seroprevalence in sika deer from different geographical locations varied from 3.13% (95% CI 0.43–5.82) to 6.73% (95% CI 3.33–10.14), but the difference was not statistically significant among the different regions (*P* > 0.05). Sika deer collected in winter (4.85%, 95% CI 2.05–7.64, *P* > 0.05) had a slightly lower HEV seroprevalence compared to sika deer collected in autumn (5.65%, 95% CI 3.83–7.46), and the difference was not significant statistically.

## 4. Discussion

There are many reports showing that HEV could infect deer all over the world (Table 2). In the present study, we surveyed the HEV seroprevalence in sika deer at 4 cities in Northern China. The overall HEV seroprevalence in sika deer was 5.43%, which was lower than that in red deer (*Cervus elaphus*) in Spain (10.4%) [27] and Yezo deer in Japan (34.8%) [26] by ELISA and in red deer in Spain (13.6%) [27] and Netherlands (15%) [22] and roe deer in Hungary (34.4%) [28] by RT-PCR but higher than that in red deer in Netherlands (5%) [22] and wild sika deer in Japan (2.6%) [25] and USA (0%) [7] by ELISA and in wild sika deer in Japan by RT-PCR (0%) [25] and Western blot (2.8%) [26] (Table 2). It is well known that due to high discordance between assays in different detection methods, the actual discrepancy is difficult to explain in the prevalence of HEV among different studies. The differences may also be due to the investigated areas that challenged the survival of HEV, the differences in sanitation, animal-welfare for deer, animal husbandry practices, and geographical and ecological factors, for example, rainfall.

Previous studies [29, 37] demonstrated the male has the higher positive rates than the female, but the present study indicates the opposite although the difference in prevalence between females and males is not statistically significant (Table 1). In different regions group, HEV seroprevalence in sika deer varied from 3.13% (5/160) to 6.73% (14/208). Sika deer in Harbin has the highest HEV seroprevalence, followed by Changchun, the third was Jilin City, and Chifeng was the last, but the difference was not statistically significant (*P* = 0.36) (Table 1). Climate, geography, degree of environment contamination with HEV, ecological conditions, feeding conditions, and animal welfares could be the reasons for the differences in HEV seroprevalence in sika deer in different cities.

In terms of the sampling seasons, HEV seroprevalence in sika deer collected in autumn was 5.65%, which was higher than that collected in winter (4.85%), but the difference was not statistically significant (*P* = 0.65) (Table 1). This may happen because in autumn the local climate was suitable for the survival of HEV and the resistance of sika deer was improved in winter in four cities. Compared with autumn, sika deer has a small range of activities and the relatively lower exposure of HEV in winter.

Sika deer are popular in China, and they are famous for medicinal value. Velvet antlers, blood, and meat are the main products from sika deer. Deer meat especially was widely recognized by most people with the improvement of living standards in Northern China. A study has shown that HEV could be transmitted from deer to humans [37], so sika deer is considered as the potential source for the spread of HEV to humans, but, in this study, we did not demonstrate that the elder have higher HEV seroprevalence than the younger [38] because the samples were collected in four cities in northern China between October 2012 and October 2013, just including autumn and winter samples, and the animals were adult sika deer, so our results may have not reflected the relationship between HEV seroprevalence and age. Moreover, the study only detected the antibodies against HEV in sika deer, and no sequence information was obtained due to limited volume of serum, so it could not reflect the true infection rate. Further studies should be conducted to get sequence information to confirm the real HEV infection rates and to determine the HEV genotypes.

## 5. Conclusion

The present investigation suggests the existence of HEV infection in sika deer, Northern China. The result may provide fundamental information for estimating the effectiveness of future measure to control HEV infection in sika deer in China and assessing the potential risk of humans infected by HEV.

## Figures and Tables

**Table 1:** Seroprevalence of hepatitis E virus (HEV) infection in sika deer (*Cervus nippon*) in different region, gender, and season by enzyme linked immunosorbent assay (ELISA).

Variable	Category	Number of examined deer	Number of positive deer	Prevalence (%) (95% CI)	*P* value	OR (95% CI)
Gender	Male	540	26	4.82 (3.01–6.62)	0.29	Reference
	Female	307	20	6.52 (3.75–9.28)		1.38 (0.76–2.51)

Region	Harbin	208	14	6.73 (3.33–10.14)	0.36	Reference
	Changchun	316	20	6.33 (3.64–9.01)		0.94 (0.46–1.90)
	Jilin City	163	7	4.29 (1.18–7.41)		0.62 (0.25–1.58)
	Chifeng	160	5	3.13 (0.43–5.82)		0.45 (0.16–1.27)

Season	Autumn	620	35	5.65 (3.83–7.46)	0.65	Reference
	Winter	227	11	4.85 (2.05–7.64)		0.85 (0.43–1.71)

Total		847	46	5.43 (3.91–6.96)		

**Table 2:** Prevalence of hepatitis E virus (HEV) infection in deer around the world.

Country	Species	Test^a^	Number of tested deer	Positive (%)	Year^b^	Reference
Poland	Red deer (*Cervus elaphus*)	ELISA	118	0	2012-2013	[21]
Poland	Roe deer (*Capreolus capreolus*)	ELISA	38	0	2012-2013	[21]
Poland	Fallow deer (*Dama dama*)	ELISA	5	0	2012-2013	[21]
Poland	Sika deer (*Cervus nippon*)	ELISA	4	0	2012-2013	[21]
Netherlands	Red deer	ELISA	38	5	2005–2008	[22]
Netherlands	Red deer	RT-PCR	39	15	2005–2008	[22]
Japan	Deer	ELISA	117	2	2003-2004	[23]
USA	Wild sika deer	ELISA	174	0	UN	[24]
Japan	Wild sika deer	ELISA	976	2.6	UN	[25]
Japan	Wild sika deer	RT-PCR	247	0	UN	[26]
Japan	Yezo deer	ELISA	520	34.8	UN	[26]
Japan	Yezo deer	WB	520	2.8	UN	[26]
Spain	Red deer	ELISA	968	10.4	2000–2009	[27]
Spain	Red deer	RT-PCR	968	13.6	2000–2009	[27]
Hungary	Roe deer	RT-PCR	32	34.4	2001–2006	[28]

^a^ELISA: enzyme-linked immunosorbent assay; RT-PCR: reverse transcription polymerase chain reaction; WB: Western blot.

^b^UN: unknown.
